# Precision Endonasal Endoscopic Surgery of the Frontal Recess Cells and Frontal Sinus Guided by the Natural Sinus Drainage Pathway

**DOI:** 10.3389/fsurg.2022.862178

**Published:** 2022-04-25

**Authors:** Zhouying Peng, Yumin Wang, Yan Fang, Yaxuan Wang, Xiang Chen, Ruohao Fan, Hua Zhang, Zhihai Xie, Weihong Jiang

**Affiliations:** ^1^Department of Otolaryngology Head and Neck Surgery, Xiangya Hospital, Central South University, Changsha, China; ^2^Otolaryngology Major Disease Research Key Laboratory of Hunan, Changsha, China; ^3^National Clinical Research Center for Geriatric Disorders, Xiangya Hospital, Central South University, Changsha, China; ^4^Anatomy Laboratory of Division of Nose and Cranial Base, Clinical Anatomy Center of Xiangya Hospital, Central South University, Changsha, China

**Keywords:** endonasal endoscopic surgery, drainage pathways, frontal recess, frontal sinus, mucoceles

## Abstract

**Background:**

The endoscopic endonasal approach to removing lesions in the nasal cavity and sinuses has become the modern first choice. However, if endoscopic surgery is performed without proper knowledge of sinus anatomy, there is a risk of residual lesions, recurrence, and even serious complications. Therefore, this article illustrates the importance of precise sinus opening guided by the natural sinus drainage pathway, using the anatomy of the frontal sinus (FS) and the frontal recess (FR) cells as an example.

**Method:**

A total of 82 sides cadaveric heads were dissected and analyzed, and the natural drainage pathways of the FR cells and FS were observed at 0°and 70°nasal endoscopic views, and the findings were summarized. The data of 79 patients who accepted endonasal endoscopic surgery (EES) guided by natural sinus drainage pathways to remove mucoceles in our department from January 2015 to January 2021 were retrospectively analyzed.

**Results:**

Two natural drainage pathways of the FR cells were discovered, identified, and named the medial pathway of the FR (MPFR) and the lateral pathway of the FR (LPFR). The 79 patients who accepted EES to remove mucoceles through the natural drainage pathways of FR cells and the FS showed significant improvement in clinical symptoms, and none of them had recurrence after surgery without serious complications.

**Conclusion:**

The EES of the FR cells and FS through the natural drainage pathways to remove the mucoceles facilitates exposure of the cells without residual lesions and without serious complications.

## Introduction

The frontal recess (FR) cells have an extremely intricate anatomical structure. Bent and Kuhn proposed the International Frontal Sinus Anatomy Classification (IFAC) system, which divides FR cells into agger nasi cells (ANC), supra agger cells, supra agger frontal cells, supra bulla cells, supraorbital ethmoid cells, frontal septal cells, and supra bulla frontal cells (1, 2). These cells are described with frontal sinus (FS) wall as a landmark. The FS is located high, and if there is a lack of proper anatomical understanding, it is easy to mistake the other FR cells as it during endonasal endoscopic surgery (EES). Improper surgery is even more likely to cause serious complications (3, 4).

Mucocele is one of the most common benign lesions occurring in the paranasal sinuses (5). The frequency of occurrence is the highest in the FS, followed by the frontoethmoidal, ethmoid, maxillary, and sphenoid sinuses. Mucoceles were first described in the early 1700s, and the understanding of their natural course and management has continued to evolve since then. A mucocele is an encapsulated, expansile collection of fluid within the paranasal sinus, which occurs due to the obstruction of the ostium of the sinus (6). Obstruction of sinus drainage is the primary pathophysiology of paranasal mucocele. Primary mucoceles can occur without any obvious etiology. Secondary mucoceles can occur as sequelae of surgery or trauma (7). Mucoceles accompanied by infection may cause more harm to the associated tissues.

Endonasal endoscopic surgery is the main treatment modality for mucoceles. Due to its specific causative factors, we believe that EES removes the lesion by precisely opening the cells through the natural sinus drainage pathways, which is undoubtedly the best option for the treatment of mucoceles. Therefore, we collected the data of patients who accepted EES to expose the FR cells and the FS guided by the natural drainage pathways and to remove mucoceles in the past 6 years at our institution. Their postoperative outcomes and complications were investigated in order to clarify the importance of exposure cells through the drainage pathways of the FR and FS.

## Materials and Methods

### Study Design and Participants

A total of 82 sides of 45 adult cadaveric heads, 41 left and 41 right (some not used due to damage), were included in this study and dissected using 0° and 70° wide-angle nasal endoscopes, and anatomical images were collected for analysis. Then, we collected the clinical data from patients who accepted precision EES exposure sinus excision of mucoceles guided by the natural drainage pathways of the FR cells and the FS in our department between January 2015 and January 2021 and were followed up.

### Brief Technical Notes

**Figure 1:**
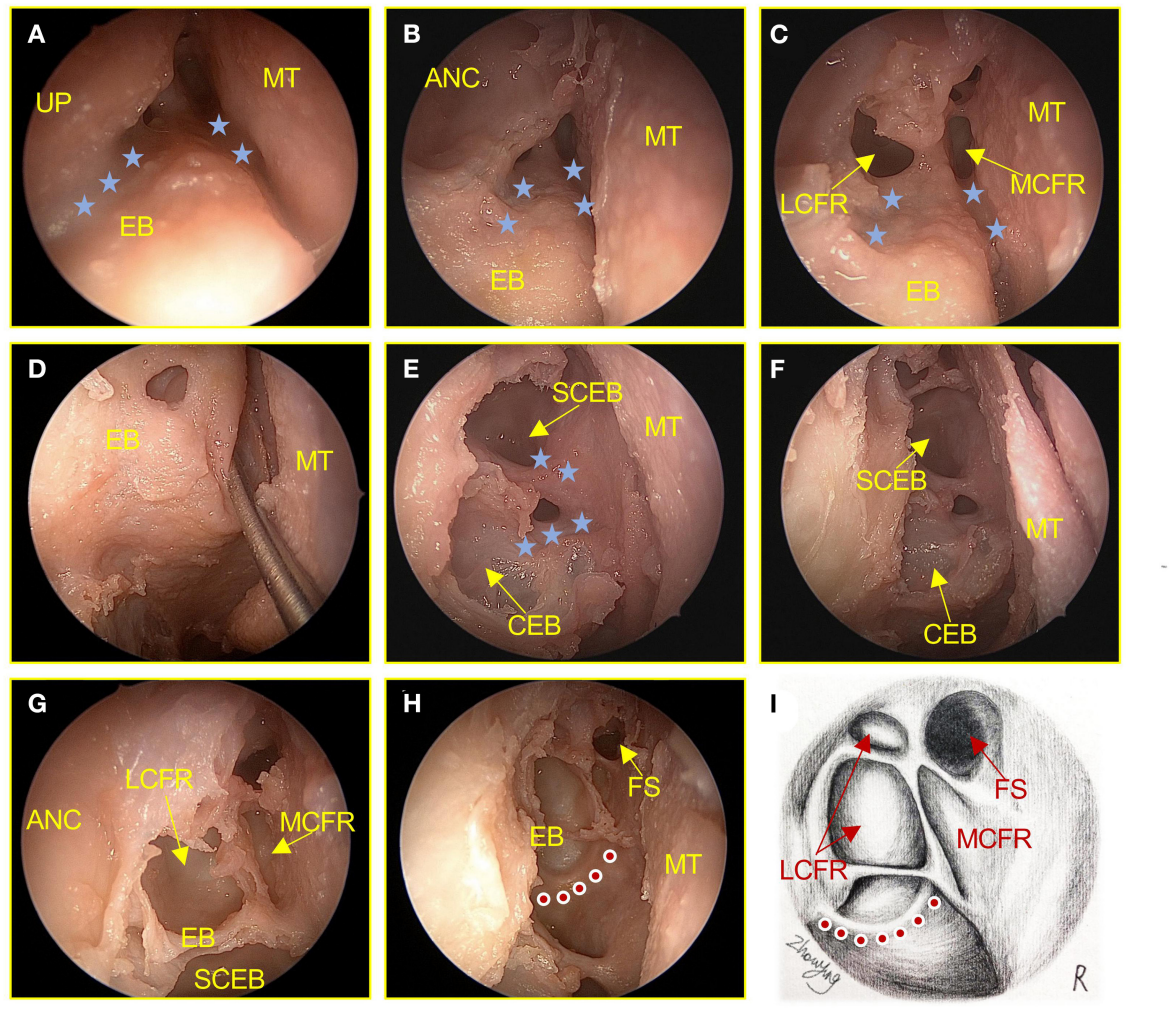
Surgical procedures of the anterior ethmoid sinus in an adult cadaver head (right). **(A)** The UP, MT, and EB of the frontal recess were examined with the aid of a 70° endoscope. The blue star shows the natural drainage pathway of the frontal recess. **(B)** The ANC was examined with the aid of a 70° endoscope. **(C)** The front-end bone plate was resected to expose the cells draining into the LCFR, and MCFR was examined with the aid of a 70° endoscope. **(D)** Drainage pathway for ethmoid bulla. **(E)** The front-end bone plate was resected to expose the cells of the ethmoid bulla. **(F–H)** Remove all bone plates to the terminal attachment to completely expose the anterior ethmoid sinus cells. The red dot shows the anterior sieve artery. **(I)** Hand-drawn anatomical drawings show the anterior ethmoid sinus cells were completely exposed. UP, uncinate process; MT, middle turbinate; EB, ethmoid bulla; ANC, agger nasi cells; LCFR, lateral cells of the frontal recess; MCFR, medial cells of the frontal recess; CEB, cells of the ethmoid bulla; SCEB, supra bulla cells of the ethmoid bulla; FS, frontal sinus.

The right nasal cavity was examined using a 0° endoscope, and then the middle and horizontal portions of the uncinate process (UP) were removed while leaving the upper portion of the UP completely intact. With the aid of a 70° endoscope, the upper portion of the UP was partially removed to fully expose the ANC, the lateral cells of the FR (LCFR), and the medial cells of the FR (MCFR). The medial suprainfundibular plate (MSIP) separates the LCFR from the MCFR, and the lateral suprainfundibular plate (LSIP) separates the ANC from the LCFR. Under 0° endoscopic view, the ethmoid bulla (EB) was exposed, and after following its drainage pathways, the supra bulla cells of the EB (SCEB) and the cells of the EB (CEB) were opened and revealed along the drainage pathway. By removing all the bone to the attachment end, the FR cells and FS can be completely opened, as shown in Figure 1.

### Statistical Analysis

Descriptive data are presented as percentages for categorical variables and means with standard deviations for parametric continuous data. Statistical analysis was performed using the GraphPad Prism Software (version. 8.4.2).

### Ethics Approval and Consent to Participate

This study was approved by the Xiangya Hospital Research Ethics Committee of Central South University (202006080). All patients provided written consent before participating in the study, which was conducted according to the Declaration of Helsinki.

## Results

### Anatomical Data of FR and FS

**Figure 2:**
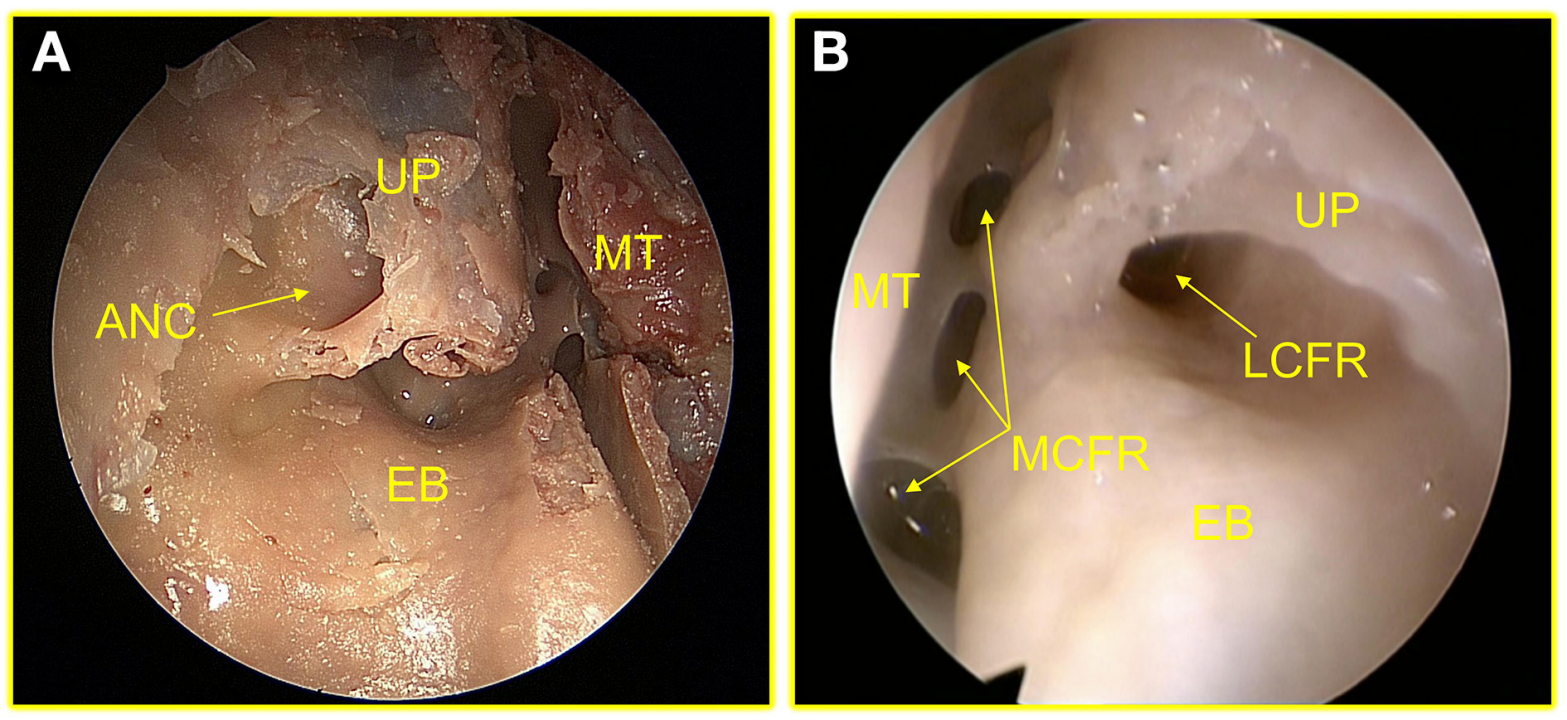
**(A)** The arrow points to the ANC. **(B)** This sample had no ANC and only LCFR. UP, uncinate process; MT, middle turbinate; ANC, agger nasi cells; EB, ethmoid bulla; MCFR, medial cells of the frontal recess.

**Figure 3:**
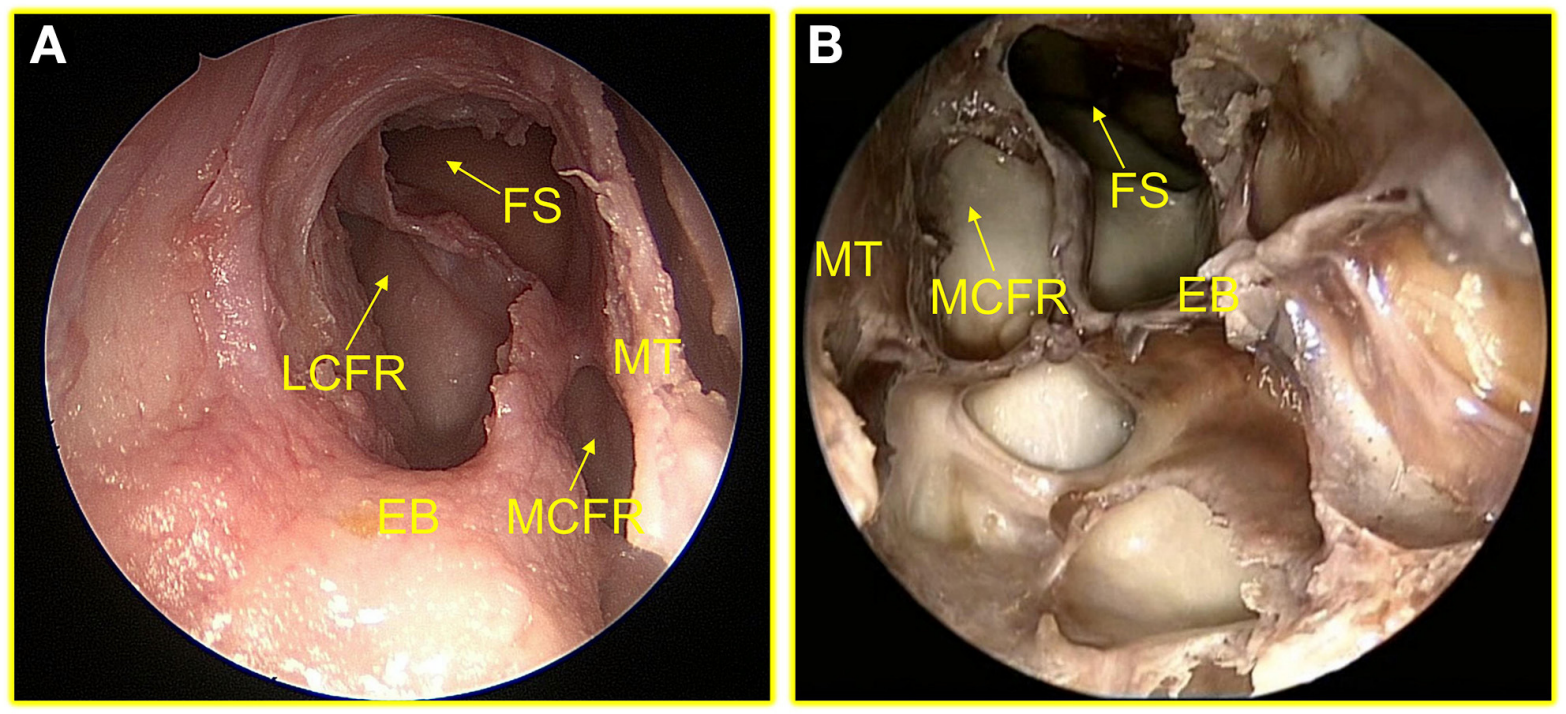
**(A)** The drainage pathway of FS is located at MPFR. **(B)** The drainage pathway of FS is located at LPFR. FS, frontal sinus; LCFR, lateral cells of the frontal recess; MT, middle turbinate; EB, ethmoid bulla; MCFR, medial cells of the frontal recess; MPFR, medial pathway of the FR; LPFR, lateral pathway of the FR.

Of the 82 sides adult cadaveric heads dissected, it was found that ANC and LCFR drained from the LPFR, but ANC was not necessarily present in 19.5% (16/82) of the specimens, as shown in Figure 2. MCFR was drained from the MPFR. The FS drainage pathway could be located in the MPFR or in the LPFR, of which 87.8% (72/82) were located in the LPFR, as shown in Figure 3.

### Population and Clinical Manifestation

This study included 79 patients who met the inclusion criteria and accepted surgical treatment for mucoceles between January 2015 and January 2021. The mean age of the patients in the study population was 49.2 years (range 20–81). There was a higher proportion of men in the study group, with 57% men and 43% women. The demographic characteristics of the patients and the etiology of mucoceles were presented in Table 1. Each patient was prescribed routine antibiotics after surgery (usually 3 days postoperatively). They were advised to use a nasal irrigator to rinse the nasal cavity two times a day after discharge, along with standardized nasal spray hormones. The patients visited our hospital for nasal cleanup approximately 2 weeks after discharge. We determined the need for a second cleanup based on the specific circumstances. The relationship between the patient's major clinical manifestations and the location of the lesion is shown in Table 2.

**Table 1:** Patients' demographic characteristics and etiology of mucocele.

**Characteristic**	***N*** **= 79**
Demographics	
Female	34(43.0%)
Male	45(57.0%)
Etiology of mucocele	
After nasal surgery	11(13.9%)
After nasal injury	5(6.3%)
Sinus occlusion without obvious causes	63(79.8%)
Location of lesion	
Frontal sinus	17(21.5%)
Frontal and ethmoid sinus	54(68.4%)
Involving the orbital cavity	8(10.1%)

**Table 2:** Principal symptoms and site of lesion in the study participants (*n* = 79).

	**Ocular symptom**	**Nasal symptom**	**Head symptom**	**Complex symptoms (≥two major symptoms)**	**No obvious symptom**
Patient number	33 (41.8%)	6 (7.6%)	25 (31.6%)	9 (11.4%)	6 (7.6%)
Site of lesion					
Frontal sinus	4	/	13	/	/
Frontal and ethmoid sinus	25	5	12	6	6
Involving the orbital cavity	4	1	/	3	/

### Prognosis

**Figure 4:**
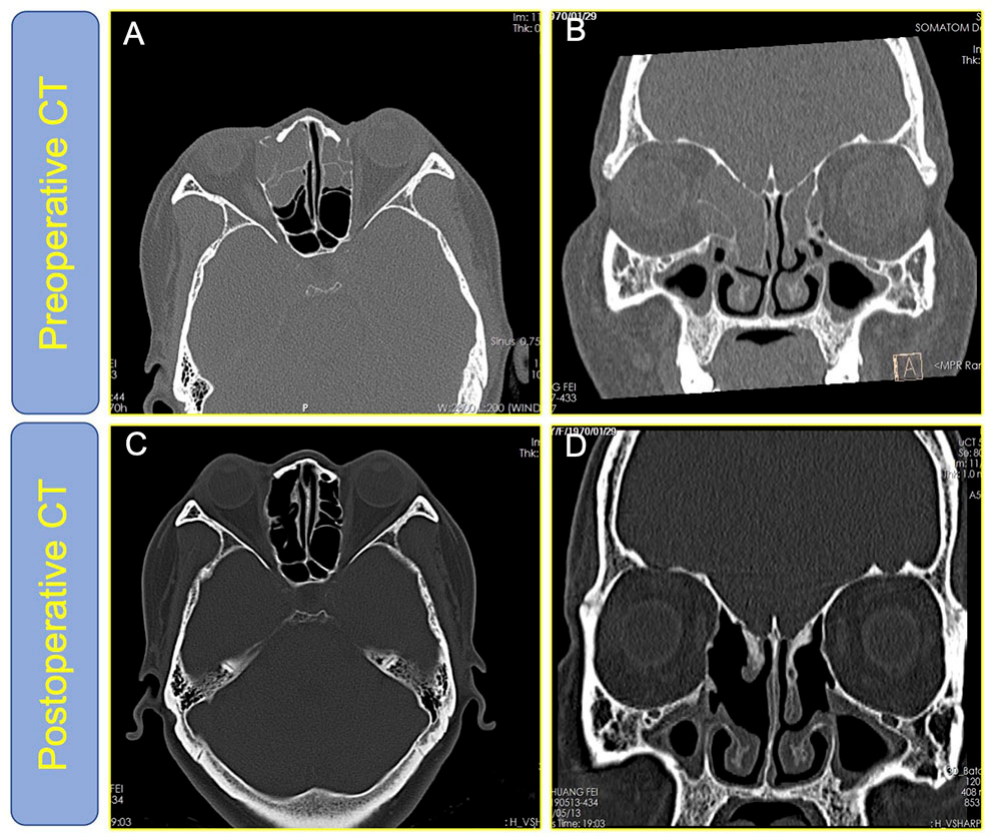
**(A,B)** The high-resolution CT of the nasal sinuses of patient #70 before surgery; **(C,D)** The high-resolution CT of the nasal sinuses of patient #70 1 year after surgery showed that all diseased sinuses were open and the disease-free sinuses are undisturbed and the nasal mucosa is well preserved.

Each patient will have a nasal endoscopy 3 months after surgery to check the opening of the sinus cavity and the epithelialization of the operation cavity. Almost every patient had a good opening of the sinus ostium after the operation, and epithelialization of the operation cavity was great. Some patients will have a repeat computed tomography (CT) examination at 12 months postoperatively, and Figure 4 shows the patient's preoperative and postoperative CT.

There was no recurrence of mucocele within the minimum follow-up period of 12 months. Only one patient developed nasal bleeding within 10 days of surgery; the remaining patients did not experience any postoperative complications. None of the patients had serious complications, such as orbital and skull base injuries.

**Figure 5:**
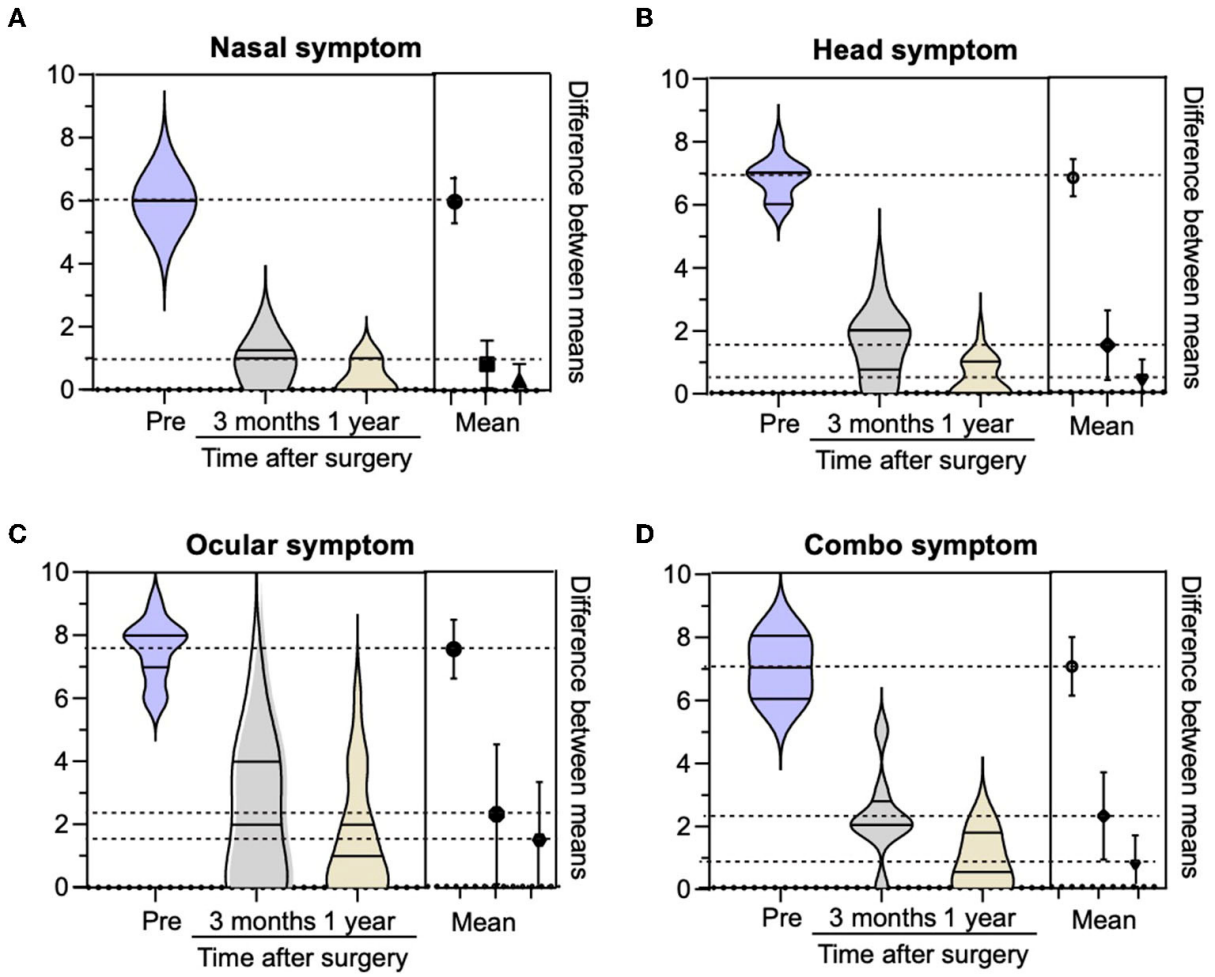
Comparison of visual analog scores (VAS) of each symptom group preoperation, 3 months after the operation, and 1 year after the operation.

We collected and analyzed the clinical symptoms and visual analog scores (VAS) of 79 patients preoperatively at 3 months and 1 year after surgery. The VAS scores at these three time-points were compared with respect to the ocular, headache and nasal symptoms, and complex symptoms (≥2 symptoms). We found that the patients' clinical symptoms improved significantly after surgery. The subjective scores of patients with preoperative head and ocular symptoms were relatively high, but the remission efficiency of the postoperative head-symptom group was significantly higher than that of the ocular-symptoms group. The preoperative symptom score of patients with nasal symptoms was lower than that of the other three groups, while the postoperative remission efficiency was higher. The postoperative symptom remission efficiency was affected by the presence of ocular symptoms in the combination symptom group. Figure 5 shows more intuitively the changes of VAS scores in preoperative and postoperative reviews of several groups of patients.

## Discussion

Endonasal endoscopic FS surgery is the most difficult procedure in nasal endoscopic sinus surgery, and its difficulty is reflected in the large variation of the FS opening, the complex anatomy of the FR, and the large number of adjacent important structures (8, 9). Therefore, endonasal endoscopic FS surgery has been an area of exploration for nasal surgeons, mainly focusing on how to find the location of the FS in a better manner. Through a long period of research and surgical development, the following views are recognized: Fridman et al. proposed to determine the location of FS drainage by the attachment of the upper part of the UP (10). While PJ Wormald advocated the modified endoscopic Lothrop procedure as an effective form of treatment in the management of complicated FS disease (11). Recently, Yoon et al. (12) have proposed “SIP” as the key anatomy for endonasal endoscopic FS surgery. Compared with the previously mentioned common procedures for endoscopic FS surgery, the Draf IIb and Draf III types of FS surgery and the “axillary flap technique” created by Draf et al. have the advantage of completely opening the FS and are suitable for patients who lack surgical anatomical landmarks after multiple surgeries (13–15). However, these techniques tend to cause stenosis owing to excessive mucosal injury in the drainage pathway and the floor of the FS, as well as excessive bone exposure. Our previous studies have concluded from anatomical studies on a large number of adult cadaveric heads. MSIP is the key landmark to identify MPFR, LPFR, and the classification of FR cells, and using the exposed natural drainage pathway as a marker instead of using the unexposed wall of FS may help to expose FR cells and FS during surgery (16). Of course, this theory needs to be supported by the results of more clinical studies.

Mucoceles are benign lesions that are commonly encountered in nasal skull base surgery. Mucoceles can also frequently appear with common clinical manifestations of malignant tumors, especially when secondary infection leads to the formation of purulent cysts, which can cause bony resorption of the sinus wall. These lesions can eventually extend into the orbit and cause vision defects, visual field defects, eyeball displacement, and other symptoms. Lesions of the ethmoid sinus and frontal sinus can even extend into the skull, causing serious complications. Therefore, active removal is essential once a lesion is detected (17, 18). EES is the current surgical treatment of choice. Obstruction of sinus drainage is the primary pathophysiology of paranasal mucocele (7). Primary mucoceles can occur without any obvious etiology. This shows that the application of our previous findings to the surgical treatment of the disease may have good results. Therefore, we summarized the clinical data of patients with mucoceles in the FR cells and FS in the last 6 years and followed them for a maximum follow-up period of 70 months. At present, none of the above patients have any significant recurrence symptoms.

Our study participants were evaluated using subjective symptom scores and CT. The results of this study showed that our precision EES exposure sinus excision of mucoceles guided by natural drainage pathways of the FR and FS prevented serious harm to the patient's nasal structures under the premise of fully alleviating the clinical symptoms. The treatment approach of sinus mucoceles or other diseases of the nasal cavity and sinuses (such as chronic rhinosinusitis, fungal sinusitis, and sinus obstructive inflammation caused by some tumors) should be guided by the following principles. The CT scan should be read carefully before surgery, and be correlated with the patient's subjective symptoms and nasal endoscopic results before determining the basic direction of surgery. These preoperative preparations can ensure better outcomes with this EES. The EES for FR cells and FS guided by the natural drainage pathways may be simpler with a lower rate of surgical injury.

## Conclusion

The EES of the FR cells and FS through the natural drainage pathways to remove the mucoceles facilitates exposure of the cells without residual lesions and serious complications.

## Data Availability Statement

The original contributions presented in the study are included in the article/supplementary material, further inquiries can be directed to the corresponding authors.

## Ethics Statement

The studies involving human participants were reviewed and approved by Xiangya Hospital Central South University. The patients/participants provided their written informed consent to participate in this study.

## Author Contributions

WJ and ZX conceived and designed the study and supervised the study. ZP, YaW, XC, RF, and YuW acquired data, searched the publications, performed the analysis, and prepared the figures and table. ZP wrote the main manuscript. YF modified the images. Manuscript corrected by HZ and YF. All authors read, reviewed, and approved the final manuscript.

## Funding

This research was funded by the National Natural Science Foundation of China (82171118), the Hunan Postdoctoral Program for Innovative Talent (2021RC2017), and the Natural Science Foundation of Hunan Province (2021JJ41027). The funders had no role in study design, data collection and analysis, decision to publish, or preparation of the manuscript.

## Conflict of Interest

The authors declare that the research was conducted in the absence of any commercial or financial relationships that could be construed as a potential conflict of interest.

## Publisher's Note

All claims expressed in this article are solely those of the authors and do not necessarily represent those of their affiliated organizations, or those of the publisher, the editors and the reviewers. Any product that may be evaluated in this article, or claim that may be made by its manufacturer, is not guaranteed or endorsed by the publisher.
